# A Fast Spatial Pool Learning Algorithm of Hierarchical Temporal Memory Based on Minicolumn's Self-Nomination

**DOI:** 10.1155/2021/6680833

**Published:** 2021-03-17

**Authors:** Lei Li, Tingting Zou, Tao Cai, Dejiao Niu, Yuquan Zhu

**Affiliations:** Department of Computer Science and Communication Engineering, Jiangsu University, Zhenjiang, China

## Abstract

As a new type of artificial neural network model, HTM has become the focus of current research and application. The sparse distributed representation is the basis of the HTM model, but the existing spatial pool learning algorithms have high training time overhead and may cause the spatial pool to become unstable. To overcome these disadvantages, we propose a fast spatial pool learning algorithm of HTM based on minicolumn's nomination, where the minicolumns are selected according to the load-carrying capacity and the synapses are adjusted using compressed encoding. We have implemented the prototype of the algorithm and carried out experiments on three datasets. It is verified that the training time overhead of the proposed algorithm is almost unaffected by the encoding length, and the spatial pool becomes stable after fewer iterations of training. Moreover, the training of the new input does not affect the already trained results.

## 1. Introduction

Neural computing is a hot topic in the field of artificial intelligence and machine learning. Hierarchical temporal memory (HTM) is a kind of machine learning technology that simulates the organization and mechanism of cerebral cortex cells as well as the information processing pipeline of the human brain. HTM is trained with a large number of time-series data and stores a large number of pattern sequences. Through the memory, HTM can predict the future inputs or detect whether the current input is abnormal according to the temporal history information.

HTM works based on sparse distributed representations (SDRs) of the inputs. Spatial pool learning algorithm (SPL) establishes the connection between the input code and the minicolumn's synapses using the Hebbian learning rule and activates the column set by a significant bit in the input code, thus realizing the SDR of input. In this process, the spatial pool is expected to maintain a certain degree of flexibility like the cerebral cortex, so several overlapping goals determine how the spatial pool operates and learns, which include the following: (1) all columns should learn to represent something useful regardless of how many columns the spatial pool has. (2) A region needs to form a sparse representation of the input, i.e., only a small percentage of the columns in the spatial pool should be “winners” for an input. (3) All columns should represent nontrivial patterns of a certain level of complexity. (4) A column should be avoided to respond strongly to many distinctly unrelated input patterns, i.e., a column should represent a limited number of input patterns, sometimes only one. (5) The spatial pool should have the ability of self-adjusting to cope with various changes, including the damage of the columns and the damage or change of sensory organs. SPL uses the boosting rule, inhibition rule, minimal threshold, large synaptic pool, and Hebbian rule to enable the dynamic configuration of the spatial pool, thus achieving the desired effect after multirounds of training. In the conventional training, the time overhead of the algorithm, the sparsity of the active minicolumns, the stability of the spatial pool, and the utilization rate of the minicolumn are the main factors to evaluate the HTM training. In SPL, a large synaptic pool has a great impact on the training time overhead, and the boosting and inhibition rules lead to the instability of the spatial pool. In this paper, we analyze the characteristics of SPL and propose a fast spatial pool learning algorithm of HTM based on minicolumn's self-nomination (SPL_SN), which includes the calculation rules of the self-nomination status of the minicolumn, the selection rule of the minicolumn based on the load-carrying capacity and level, and the synapse adjustment rule based on compressed encoding. The prototype of the proposed algorithm is tested on three kinds of datasets, and four criteria are used to evaluate the time overhead, the stability of the spatial pool, the sparsity of active minicolumns, and the utilization rate of all columns. The main contributions are as follows:

1. SPL_SN reconstructs the structure of the spatial pool and adds the matching input information to each minicolumn. Based on the load-carrying capacity of the minicolumn, i.e., the utilization degree of the minicolumn, the SPL_SN can make the selected minicolumns sparsely distributed in the spatial pool.
2. Different from SPL that uses overlapping values to select minicolumns, SPL_SN uses the overlapping values to calculate the self-nomination status of the minicolumn. The self-nomination status and the load-carrying capacity of the minicolumn can improve the utilization rate of minicolumns in the spatial pool.
3. Different from SPL using the Hebbian rule to adjust the link between the input and minicolumn, SPL_SN designs a synaptic adjustment rule based on compressed encoding that can reduce the training time overhead. The learning of new input does not affect the trained information in the minicolumn, ensuring a dynamic adaptability of HTM and improving the efficiency of HTM training and the stability of the spatial pool.
4. The prototype of SPL_SN is implemented in the HTM open-source framework, and different types of datasets are used for test and analysis. It is verified that the algorithm not only meets the flexibility of the spatial pool but also improves the training efficiency and stability of the spatial pool.

## 2. Related Works

**Figure 1:**
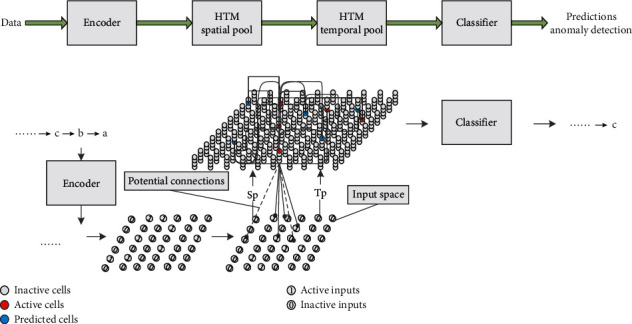
HTM workflow. It describes that, after the input is encoded, trained in the space pool, and trained in the time pool, the subsequent prediction can be made according to the memory.

Neural networks (NNs) have achieved great success in many fields, e.g., classification [1], recognition [2], prediction, and control. In order to further improve their performance and efficiency, NNs are also exploring in different directions, such as using dendritic neurons to build networks [3] and fusing with other neural networks [4]. HTM is a new artificial neural network model based on Jeff Hawkins' memory prediction framework [5, 6], and its main workflow is shown in Figure 1. The network space of HTM is described as a region composed of multiple columns. Different regions have hierarchical relationships. There are many cells on a minicolumn. The cells on each minicolumn share a proximal dendrite that is used to receive input stimuli. Cells on the minicolumns have many branches of distal dendrites, which can be used to build relationships with other cells [7, 8]. After the encoder, the input is transformed into a binary vector of equal length, and the number of components with 1 in the vector is equal. Through SPL, the vector activates a few minicolumns to represent the input data. The temporal pool learning algorithm (TPL) selects some cells on the activated minicolumn to express the input location information and establishes dendrite branches on these cells to construct the correlation between inputs. The sequence is learned through SPL and TPL to form memory in the network space of HTM. When the input sequence is consistent with the memory sequence, the HTM can predict the next content based on memory [9]. Two core algorithms of HTM, SPL and TPL, are used to construct the space invariant [10] and time invariant [11] features of input sequences, which are the key technologies for HTM to distinguish similar sequences.

With its advantages in time-series data processing, HTM has been used to deal with a variety of learning tasks in the field of time-series data. For example, HTM can be used to identify traffic anomalies in computer networks [12, 13], to detect abnormal behavior of website users [14], to process biological signals, to predict position in the background of smart home [15], to identify abnormal trajectories of the constructed geospatial travel pattern [16], to detect anomalies in stock trading data [17], to detect the optic nerve abnormalities in retinal image [18], to detect the online sequential attack [19], to establish IT event response system, and so on. These applications have achieved good results.

For SPL, Ahmad and Hawkins give the characteristic description of SDR and the expression form of information in the spatial pool [20], which provides theoretical guidance for the following research. Lattner uses the vector to express and study the spatial pool. He proposes the formula of calculating the overlapping value and rules of learning [21]. Byrne describes these formulas by a matrix [22], which provides the mathematical basis for the derivation of rules. Leake et al. study and discuss the influence of initialization parameters on spatial pool calculation [23]. Mnatzaganian et al. give a comprehensive mathematical framework of spatial pool [24], so that the follow-up scholars can understand the core characteristics of SPL and improve the algorithm from a mathematical point of view. These studies quantitatively analyze the existing HTM and provide guidance for the adjustment of training parameters. If the application has a large input space, the large synaptic pool maintained by the spatial pool causes intolerable training time overhead, and the boosting and inhibition rules make the spatial pool unstable. Although the training results of the spatial pool can be stabilized by closing the training, the operation will prevent the new inputs from entering the training process.

## 3. The Proposed Algorithm

### 3.1. Analysis of the Current Spatial Pool Learning Algorithm

SPL mainly uses the overlapping value to activate the minicolumn and dynamically adjusts the synaptic value of the minicolumn to strengthen their relationship. By boosting factors, the less-active minicolumn can also win the competition, and each minicolumn in the space pool can participate in representing the input. By the inhibition radius, the activated minicolumns will not concentrate together, which ensures that the winning set of minicolumns is sparsely scattered in the spatial pool. By adjusting the minicolumn's synapses according to the Hebbian competition rule, the training results of the spatial pool tend to be stable after several iterations.

However, the minicolumn activation rule and minicolumn synaptic adjustment rule make the training results of the spatial pool difficult to stabilize, and the learning efficiency of the algorithm is low. Next, the limitations of these two rules are analyzed in terms of the stability of the spatial pool and the efficiency of the current algorithm.

#### 3.1.1. The Limitations of the Minicolumn Activation Rule

SPL uses the following equation to calculate the overlap between the minicolumn and the input.

(1) $$\begin{array}{c} o_{i}=b_{i}\sum_{j\in \Pi_{i}}w_{ij}x_{j}, \end{array}$$

where *w*_*ij*_ indicates whether the connection between the *i*-th minicolumn and the *j*-th component in the input code is connected, *x*_*j*_ is the value of the *j*-th component in the input code, *b*_*i*_ is the boosting factor of the *i*-th minicolumn, and Π_*i*_ is the receptive domain of the *i*-th minicolumn.

In order to activate fewer minicolumns to participate in the input expression, the algorithm takes the overlapping value multiplied by the minicolumn's boosting factor as the competition basis. The boosting factor is calculated according to the activation frequency of the minicolumn in the statistical period and the average activation frequency of all minicolumns within the inhibition radius. ${\bar{a}}_{i}^{t}$ refers to the activation frequency of the *i*-th minicolumn at time *t*. It can be calculated with the following equation. *T* is the statistical period.

(2) $$\begin{array}{c} {\bar{a}}_{i}^{t}=\frac{T-1\times {\bar{a}}_{i}^{t-1}+{\bar{a}}_{i}^{t}}{T}, \end{array}$$

${\bar{a}}_{i}^{t}$ refers to the average active frequency of all minicolumns in the inhibition radius at time *t*. It can be calculated from the following equation, where *N*_*i*_ is the set of all minicolumns within the inhibition radius of the *i*-th minicolumn.

(3) $$\begin{array}{c} {\bar{a}}_{i}^{t}=\frac{1}{\left|N_{i}\right|}\sum_{j\in N_{i}}{\bar{a}}_{j}^{t}. \end{array}$$

Using these two mean values, the boosting factor of the minicolumn can be calculated by the following equation, and *β* is the adjustment factor.

(4) $$\begin{array}{c} b_{i}=e^{-\beta \left({\bar{a}}_{i}^{t}-{\bar{a}}_{i}^{t}\right)}. \end{array}$$

The value of the boosting factor will increase greatly if the minicolumn is not activated for a long time. In this case, the minicolumn with lower activation frequency can be activated by the following equation:

(5) $$\begin{array}{c} a_{i}^{t}=\left\{\begin{array}{cc} 1, & \text{if }o_{i}\geq ZV_{i},k\text{ and }o_{i}\geq \theta_{\text{stim}},\\ 0, & \text{otherwise,} \end{array}\right) \end{array}$$

where *a*_*i*_^*t*^ indicates whether the *i*-th minicolumn is activated at time *t*. *V*_*i*_ is the set of overlapping values of minicolumns, and these minicolumns are within the inhibition radius of the *i*-th minicolumn. *ZV*_*i*_, num returns the *k*-th value in *V*_*i*_ according to the overlapping values, and *k* is the number of minicolumns to be activated. *θ*_stim_ is the minimum threshold to activate the minicolumn.

First, the code length determines the number of the minicolumn's synapses and affects the size of minicolumn's receptive domain. The long encoding will increase the workload of calculating the overlapping values and adjusting synaptic persistence. The computational efficiency of the current spatial pool learning algorithm is greatly affected by the code length. Secondly this minicolumn activation rule will cause the spatial pool unstable, especially when the input training space is small and the spatial pool capacity is large. This is because the boosting strategy ensures that all minicolumns participate in expressing the input while ignoring the need for individual input to be stably expressed. For example, if only one input *X* is trained and the overlap between *X*'s code and *y*_*i*_ is greater than the *k*-th value within *N*_*i*_ = {*y*_1_, *y*_2_,…, *y*_*i*_, *y*_i+1_,…, *y*_i+*m*_ }, then *y*_i_ will be activated in the previous training. As the training process goes on, other minicolumns are seldom activated, and their boosting factors will become very large. Then, one of them will win in the competition and be activated. This makes the minicolumn set activated by *X* unstable, which leads to ineffective distal synaptic connections in the temporal pool.

#### 3.1.2. The Limitation of Minicolumn Synaptic Regulation

When a minicolumn set is activated by the input, SPL will adjust the permanence of the proximal synapses on the minicolumns according to the Hebbian rule, which can be described as follows:

(6) $$\begin{array}{c} D_{ij}=D_{ij}+p^{+}D_{ij}^{{}^{\circ}}x_{j}-p^{-}D_{ij}^{{}^{\circ}}\left(1-x_{j}\right),\;\text{if }i\in W^{t}, \end{array}$$

where *p*^+^ represents the increment of permanence and *p*^−^ represents the decrease of permanence. *D*_*ij*_ represents the permanence of the connection between the *i*-th minicolumn and the *j*-th component in the input code. *x*_*j*_ is the value of the *j*-th component in the input code, and *W*^*t*^ is the set of active minicolumns at time *t*.

Usually, a minicolumn is involved in the expression of multiple inputs. The regulation of minicolumn synapses not only enhances the expression ability of the minicolumn for the current input but also reduces the expression ability for other inputs. If a trained input does not participate in SPL for a long time, it will lose the ability to activate the minicolumns. We can call the process of forgetting. Forgetting has its significance in the biological system, such as reducing the memory of uncommon concepts, reducing energy consumption, and highlighting the cognition of recent learning content. However, in the HTM model, forgetting in the spatial pool will bring great cost to subsequent learning. If the forgotten input enters SPL again, it will activate a new minicolumn set, which leads to an unstable expression of a given input. In this way, what the temporal pool has learned becomes meaningless or unpredictable. Therefore, the forgetting mechanism is not suitable for SPL, but it is very useful for TPL of HTM.

The Hebbian rule adjusts the spatial pool dynamically to a stable state after iterative training. If a new input destroys much information stored in the current HTM, the spatial pool needs many iterations of training to reach a new stable state. It will affect the efficiency and effectiveness of HTM.

### 3.2. The Self-Nominations of the Minicolumn

In SPL, the minicolumn has two states according to the input stimulus, *active* and *inactive*. However, in SPL_SN, the minicolumn is given three self-nomination states based on the overlapping values, the *same*, *similar*, and *distinct*. The status indicates the willingness of the minicolumn to participate in the expression of the current input. Then, through the spatial pool, the appropriate minicolumns are selected to activate in different states to express the input. In doing so, the spatial pool is given the decision to activate the minicolumns. The spatial pool can combine the statistical information of the minicolumn to select the minicolumn more reasonably and improve computational efficiency.

The overlapping value between the minicolumn and the input is only used to determine the self-nomination state of the minicolumn, which can be calculated by the following equation:

(7) $$\begin{array}{c} o_{i}=\sum_{j\in \Pi_{i}}w_{ij}x_{j}. \end{array}$$

According to the overlap value, the minicolumn can be given three different self-nomination states by the following equation:

(8) $$\begin{array}{c} \text{state}\left(i\right)=\left\{\begin{array}{cc} \text{same,} & \text{if }o_{i}>o_{\text{threshold}},\\ \text{similar,} & \text{if }\frac{o_{\text{threshold}}}{2}\leq o_{i}\leq o_{\text{threshold}},\\ \text{distinct,} & \text{if }o_{i}<\frac{o_{\text{threshold}}}{2}, \end{array}\right) \end{array}$$

where *o*_threshold_ represents the threshold at which the minicolumn can be activated and state(*i*) expresses the recommendation state of the *i*-th minicolumn under the current input training environment. The value of state(*i*) can be the *same*, *similar*, or *distinct*. If state(*i*) is the *same*, it means that the minicolumn can be activated by the current input, and a certain input that the minicolumn has participated in is extremely close to or even the same as the current input. If state(*i*) is *similar*, it means that the minicolumn overlaps with the current input, but it has not been activated. The content of a certain input that the minicolumn has participated in is similar to the current input. If state(*i*) is *distinct*, it means that the minicolumn overlaps less with the current input, and all inputs that the minicolumn has participated in are quite different from the current input.

According to state(*i*), the minicolumn can be divided into three levels, which represent the similarity between the learned content and the current input, that is, the different willingness to participate in the current input. Based on this, the minicolumn selection algorithm can improve the utilization of every minicolumn.

### 3.3. The Minicolumn Activation Rule

To improve the minicolumn section for the input, we reconstruct the spatial pool's structure and more information of the minicolumn will be used. The minicolumn not only calculates the degree of willingness to participate in the current input, i.e., the self-nomination state, but also saves the content and the number of different inputs that have been participated in through the previous learning process. This information is called the load-carrying capacity of the minicolumn, which can be used as a measure of the utilization degree of the minicolumn. By fully utilizing the self-nomination status and load-carrying capacity of the minicolumn, the appropriate minicolumn set can be selected to express the current input. Suppose that *a*_*n*_ minicolumns are used to express the input in the spatial pool and the minicolumn sets of adjacent inputs overlap at most *o*_*n*_ minicolumns. The idea of the fast training algorithm is described as follows:

1. The current self-nomination status is calculated according to the input.
2. For minicolumns with state(*i*)=*same*, the algorithm selects the minicolumns containing the current input values to activate and count them as *n*_1_. If *n*_1_ ≥ *a*_*n*_, it returns this set of active minicolumns for the input. Otherwise, the *n*_2_ minicolumns with the lowest load-carrying capacity are randomly selected from the remaining minicolumns for activation, and *n*_2_ takes the maximum value that satisfies the constraint *n*_1_+*n*_2_ ≤ *o*_*n*_. Then, the algorithm updates the load-carrying information of the *n*_2_ minicolumns, saves the current input on the minicolumns, and increases the number of inputs that the minicolumn participates in by 1.
3. For the minicolumns with state(*i*)=*similar*, *n*_3_ minicolumns with the lowest load-carrying capacity are randomly selected to activate, and *n*_3_ takes the maximum value that satisfies the constraint *n*_1_+*n*_2_+*n*_3_ ≤ *o*_*n*_. Then, the algorithm updates the load-carrying information of the *n*_2_ minicolumns, saves the current input on the minicolumns, and increases the number of inputs that the minicolumn participates in by 1.
4. For minicolumns with state(*i*)=*di*  *stinct*, *a*_*n*_ − *n*_1_ − *n*_2_ − *n*_3_ minicolumns are selected to activate. Then, the algorithm updates the load-carrying information of these minicolumns, saves the current input on the minicolumns, and increases the number of inputs that the minicolumn participates in by 1.

SPL_SN activates a predetermined number of minicolumns with state(*i*) being the same or similar, which ensures that the training results of the spatial pool satisfy the characteristic that the minicolumn sets of similar input have a certain overlap. The random minicolumn selection strategy ensures that the set of active minicolumns can be sparsely scattered in the spatial pool and improves the utilization rate of the minicolumns as much as possible. The *a*_*n*_ limits the number of activated minicolumns to ensure the convergence of the algorithm.

### 3.4. The Synaptic Adjustment Rule

There is a mapping between the minicolumn set activated by SPL_SN and the current input. Therefore, it is necessary to add synaptic connections to the input to maintain the mapping. How the active minicolumn increases the synaptic connection is shown in the following equation:

(9) $$\begin{array}{c} W_{active\_i}=W_{active\_i}\cup^{}\text{encoder}\left(\text{CurrentInput}\right), \end{array}$$

where *active*_*i* is the number of active minicolumns selected by the algorithm for the current input, *W*_*active*_*i*_ represents the connected synapses of the minicolumn. CurrentInput is the content of the current input, and encoder(CurrentInput) is the compressed code of the current input.

SPL is based on the input distribution code. The rule of the encoding is usually to express the input with a fixed-length binary vector, in which the number of components with value 1 is small and fixed. This encoding rule scatters the input characters to each component with value 1, which can be called 1-component. By encoding similar inputs with a certain number of overlapped components, the influence of noise on the input can be reduced and the robustness of the system can be improved. In the training process of the spatial pool, the number and position of the 1-components are the factors to calculate the overlapping values and adjust the synapse. Therefore, we propose an effective compressed encoding method, which expresses scalars with shorter vectors.

For any scalar *i*, the corresponding encoding rule can be given as shown in the following equation:

(10) $$\begin{array}{c} s\left(i\right)=\left(w-k\right)\times i, \end{array}$$

(11) $$\begin{array}{c} \text{encoderi}=\left\{s\left(i\right),s\left(i\right)+1,s\left(i\right)+2,\ldots ,s\left(i\right)+w\right\}, \end{array}$$

where *w* represents the number of 1-components in the code, *k* represents the overlap number of 1-components in adjacent scalar coding, *s*(*i*) represents the starting position of 1-components in input coding, and encoderi is the compressed code of scalar *i*.

In the input compressed encoding, a longer binary vector can be represented as the position vector of 1-components, and the length of the uncompressed code is hidden. Therefore, no matter what the input value is, the length of the compressed code is fixed and short. This encoding rule can improve the efficiency of calculating the overlapping values. When the input code is superimposed on the minicolumn, it shows that the minicolumn has participated in the expression of the input, so that the spatial pool can express the input with a relatively fixed set of minicolumns through fewer rounds of training. Moreover, when a new input enters the training and modifies the connection of the minicolumn's synapses, the connection among the existing synapses is not adjusted, so the trained result will not be affected. Although the adjusting rule shown in equation (11) may result in a large overlap between the minicolumn and the uncorrelated input code, the minicolumn will not be activated because the minicolumn's load-carrying capacity does not have the input information. Thus, different inputs can be mapped into different minicolumn sets.

### 3.5. The Flow of SPL_SN

The flow of SPL_SN is shown in Table 1.

SPL_SN modifies the rules of SPL. It calculates the self-nomination states of the minicolumns based on the overlapping values to express the willingness of the minicolumns to represent the input and activates minicolumns based on their self-nomination states and load-carrying capacity. The minicolumn selection rule is dominated by the spatial pool, so it can make full use of the minicolumns and improves the efficiency of spatial pool learning. The compressed encoding algorithm uses the position of 1-components to reconstruct the code which simplifies the expression of the code and improves the efficiency of the query. When learning the new inputs, the synaptic adjustment rule based on compressed encoding does not affect the trained information in the minicolumns, which improves the efficiency of training and the stability of the spatial pool.

## 4. Evaluation and Analysis

Built upon the Numenta's open-source HTM code, a prototype system of SPL_SN is implemented. Three datasets are used to evaluate the SPL and SPL_SN.

### 4.1. Datasets and Evaluation Metrics

In this paper, three datasets are used to evaluate SPL and SPL_SN. The first two datasets are constructed artificially using the integer number between 1 and *N* as the input space. Relative to the spatial pool settings, *N* takes 10 and 5000 for the smaller input space and larger input space. The last dataset is the time-period statistics of New York taxi passengers, which is constructed by choosing the number of passengers every 30 minutes between July 1, 2014, and October 13, 2014, in total 5,000 data.

We train SPL and SPL_SN with the three datasets separately and analyze the advantages and disadvantages of the algorithm through the result of each training round. The evaluation criteria are listed below:

(1) Time overhead: for the same input space and different length of code, the computation efficiency of the algorithm is evaluated by the time overhead for one round of training.
(2) Stability of the spatial pool: it includes two meanings. The first is to describe whether the spatial pool can express the input with a fixed set of minicolumns. The second is to describe whether the new input will affect the trained results. The mean stability (MS) is used to evaluate the stability of the spatial pool. Assume that the set of minicolumns corresponding to input *i* after the *k*-th round is *C*_*i*_^*k*^; then, the stability of input *i* after the *k*-th round (*S*_*i*_^*k*^) is calculated with the following equation:
  (12) $$\begin{array}{c} S_{i}^{k}=\left\{\begin{array}{cc} 1, & \text{if }C_{i}^{k-1}==C_{i}^{k},\\ 0, & \text{otherwise}. \end{array}\right) \end{array}$$
With the stability of each input (*S*_*i*_^*k*^), the mean stability (MS) of the spatial pool after the *k*-th round is calculated using the following equation, where *M* is the number of inputs:
  (13) $$\begin{array}{c} \text{MS}=\frac{1}{M}\sum_{i=1}^{M}S_{i}^{k}. \end{array}$$
(3) The sparsity of input representation: it describes whether a spatial pool can express the input with a relatively fixed number of minicolumns. After each round of training, we count the number of active minicolumns for each input and calculate their mean (*E*_sparse_) and standard deviation (*σ*_sparse_). *E*_sparse_ and *σ*_sparse_ can evaluate the sparsity of the input representation. These two values are calculated using the following equation, where *M* is the number of inputs:
  (14) $$\begin{array}{c} E_{\text{sparse}}=\frac{1}{M}\sum_{i=1}^{M}\left|C_{i}^{k}\right|, \end{array}$$
  (15) $$\begin{array}{c} \sigma_{\text{sparse}}=\sqrt{\overset{M}{\underset{i=1}{\sum }}\frac{{\left(\left|C_{i}^{k}\right|-E_{\text{sparse}}\right)}^{2}}{M}}. \end{array}$$
(4) The utilization of minicolumns: it describes whether the minicolumns can be activated more evenly. The standard deviation of the minicolumn activation frequency is used to describe this indicator. After the *k*-th round, the activation frequency of minicolumn *j* (*P*(*j*)) is calculated by the following equation, where *ac*_*j*_^*i*^ indicates whether the input *i* activates the minicolumn *j* and *M* is the number of inputs:

(16) $$\begin{array}{c} P\left(j\right)=\frac{1}{M}\sum_{i=1}^{M}ac_{j}^{i},\;ac_{j}^{i}=\left\{\begin{array}{cc} 1, & j\in C_{i}^{k},\\ 0, & \text{otherwise,} \end{array}\right). \end{array}$$

The average activation frequency of all minicolumns (*E*_frequency_) can be calculated by the following equation, where *N* is the number of minicolumns:

(17) $$\begin{array}{c} E_{\text{frequency}}=\frac{1}{N}\sum_{i=1}^{N}P\left(j\right). \end{array}$$

The standard deviation of minicolumn activation frequency (*σ*_frequency_) can be calculated by the following equation:

(18) $$\begin{array}{c} \sigma_{\text{frequency}}=\sqrt{\overset{N}{\underset{i=1}{\sum }}\frac{{\left(P\left(j\right)-E_{\text{frequency}}\right)}^{2}}{N}}. \end{array}$$

### 4.2. Testing Environment

The parameters of the spatial pool are shown in Table 2.

We have implemented the algorithm using Java in the Windows 10 operating system, and the program runs on a PC with an Intel core tm i7-7500u 2.9 GHz CPU processor and 8 GB memory.

### 4.3. Results and Analysis

#### 4.3.1. The Artificial Dataset with Small Input Space

The input space is constructed by numbers from 1 to 10. The coding length is set to 5000, 20000, and 40000, respectively. The two algorithms train all inputs for 10 rounds with different code lengths. Then, we collect and compare the results after each round.

**Figure 2:**
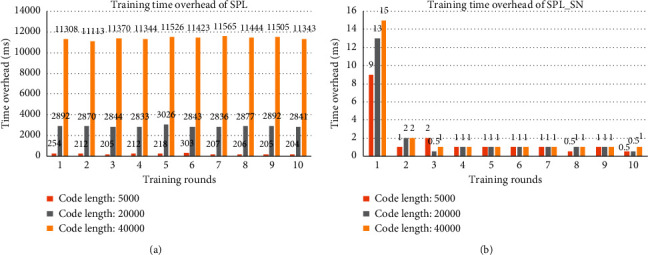
The training time overhead of two algorithms. Each column in the graph represents the training time overhead of the dataset after a round of training with different code lengths. (a) The results trained by SPL. (b) The results trained by SPL_SN.

Figure 2 shows the training time overhead in each round with different coding lengths. It can be seen that, with the increase of code length, the time overhead of SPL increases rapidly, while the time overhead of SPL_SN is much lower and almost does not increase. This is because, in SPL, the increase of code length increases the number of the minicolumn's synapses and also affects the size of the receptive domain, which increases the workload of calculating the overlapping value. In SPL_SN, the length of the compressed code is fixed and short, and the number of the minicolumn's synapses which are composed of a small number of input codes is small, so the workload of calculating overlapping values is low and hardly changed. It shows that the time cost of SPL_SN is hardly affected by the code length.

**Figure 3:**
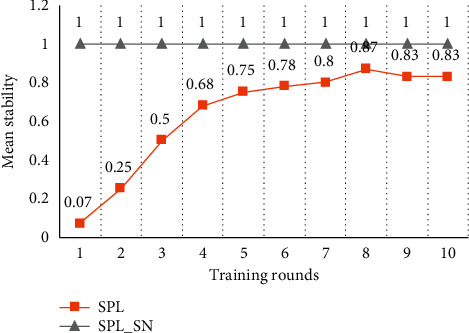
Comparison of stability in small input space. Each node in the graph represents the proportion of inputs which SDRs have not changed in the whole dataset compared with the results of the previous round of training. The dataset was trained for 10 rounds using different algorithms.

Figure 3 shows the stability of the spatial pool. When SPL trains data with small input space, the stability of the spatial pool tends to increase with more training rounds. However, the spatial pools cannot be stabilized completely because of the boosting and inhibition rules. Therefore, SPL suggests that the learning function should be turned off after certain rounds of training. This is to avoid that the instability of the spatial pool may introduce invalid distal synapses to the temporal pool. However, turning off the learning function will make the spatial pool unable to deal with the new inputs. SPL_SN maintains the high stability of the spatial pool. This is because the synaptic adjustment rule based on compressed encoding enables the proximal synapses of the minicolumn to quickly establish and solidify the corresponding mapping with the input code, to express the input with a relatively stable minicolumn set. Each subsequent training can be regarded as training on a new input relative to the trained content. If the new input training does not affect the trained content, it shows that the training algorithm has a strong anti-interference performance. SPL_SN cannot change the trained content in each training, which shows that the algorithm has a high anti-interference performance.

**Figure 4:**
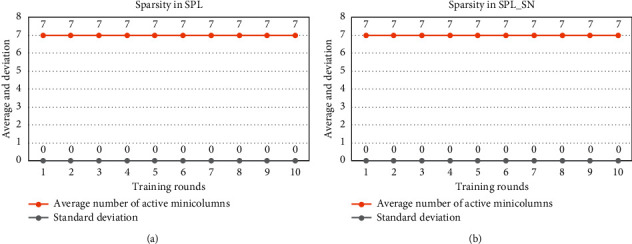
Comparison of sparsity in small input space. (a) After each round of TPL training, we calculate the mean and standard deviation of the number of activated minicolumns in each input to express the sparsity of the spatial pool. (b) After each round of TPL_SN training, we also count such indicators. The dataset was trained for 10 rounds using different algorithms.

Figure 4 shows the results on the sparsity of the input representation. SPL_SN, like SPL, is able to express the input with a sparse minicolumn set according to the parameters.

**Figure 5:**
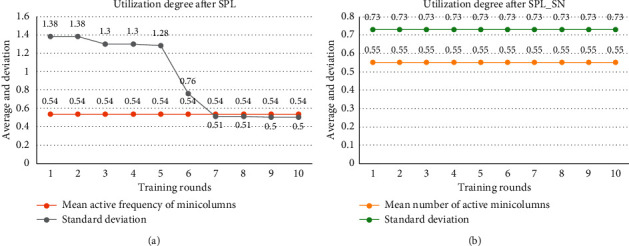
Comparison of utilization degree of the minicolumn in small input space. (a) After each round of TPL training, we calculate the mean and standard deviation of the activation frequency of each minicolumn to express the utilization degree of the minicolumn. (b) After each round of TPL_SN training, we also count such indicators. The dataset was trained for 10 rounds using different algorithms.

Figure 5 shows the minicolumn utilization. Because of the small input space, the results of the two algorithms cannot make all minicolumns participate in the input expression. In the early training of SPL, the activated minicolumns are in a small range of the spatial pool. With the boosting and inhibition rule, the activated minicolumns gradually scatter in the spatial pool. SPL_SN can express the input with a more dispersed set of minicolumns in the first round of training.

#### 4.3.2. The Artificial Dataset with Larger Input Space

The input space is constructed by numbers from 1 to 5000. The code length is set to 5000, 20000, and 40000, respectively. The two algorithms train all inputs for 10 rounds with different code lengths. Then, we collect and compare the results after each round.

**Figure 6:**
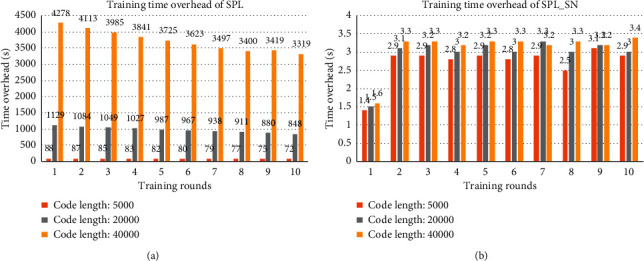
The training time overhead of two algorithms. Each column in the graph represents the training time overhead of the dataset after a round of training with different code lengths. (a) The results trained by SPL. (b) The results trained by SPL_SN.

Figure 6 shows the training time overhead in each round with different coding lengths. It also can be seen that, with the increase of code length, the time cost of SPL increases rapidly, and the time cost of SPL_SN is lower and almost does not increase. Compared with the previous dataset, the training time overhead of this dataset is much higher because the number of inputs is much more than that of the previous dataset. It shows that the time overhead of SPL is affected by the size of input space and code length, while the time overhead of SPL_SN is only affected by the size of input space and is hardly affected by the length of the code.

**Figure 7:**
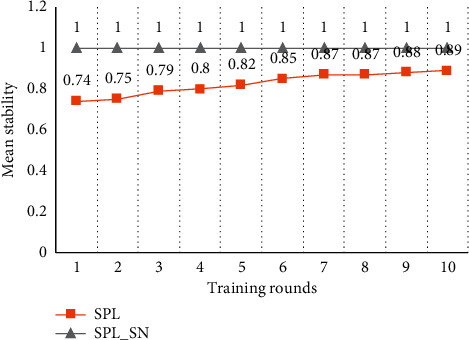
Comparison of stability in larger input space. Each node in the graph represents the proportion of inputs which SDRs have not changed in the whole dataset compared with the results of the previous round of training. The dataset was trained for 10 rounds using different algorithms.

Figure 7 shows the test results on the stability of the spatial pool. When SPL trains input data with a larger input space, the spatial pool can be more stable after the first round of training compared to the result of a small input space. This is because all minicolumns participate in the expression of inputs and the activation frequencies of the minicolumns are similar. Therefore, the probability of minicolumn synaptic adjustment is reduced and the spatial pool is relatively more stable. However, due to the boosting and inhibition rules of SPL, the spatial pool cannot achieve a completely stable state even with more training rounds. SPL_SN has established the mapping between the input and minicolumn set in the first round of training, and the subsequent training process does not change the learned content, so SPL_SN can keep the desired stability of the spatial pool.

**Figure 8:**
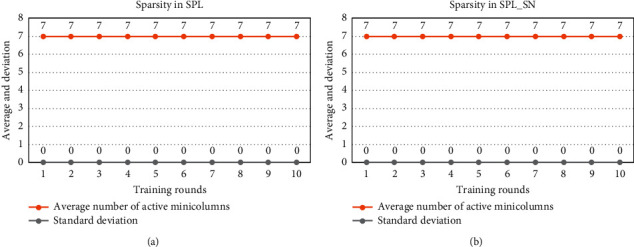
Comparison of sparsity in larger input space. (a) After each round of TPL training, we calculate the mean and standard deviation of the number of activated minicolumns in each input to express the sparsity of the spatial pool. (b) After each round of TPL_SN training, we also count such indicators. The dataset was trained for 10 rounds using different algorithms.

Figure 8 shows the sparsity of the input representation. SPL_SN, like SPL, is able to express the input with a sparse minicolumn set according to the parameters.

**Figure 9:**
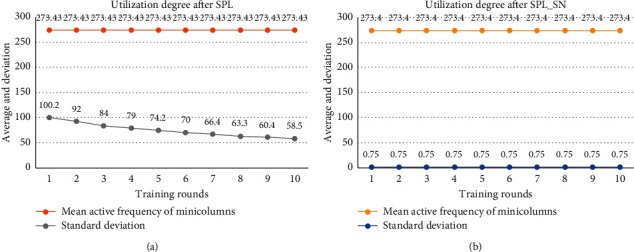
Comparison of the utilization degree of the minicolumn in larger input space. (a) After each round of TPL training, we calculate the mean and standard deviation of the activation frequency of each minicolumn to express the utilization degree of the minicolumn. (b) After each round of TPL_SN training, we also count such indicators. The dataset was trained for 10 rounds using different algorithms.

Figure 9 shows the minicolumn utilization. For the large input space, all minicolumns in the spatial pool participate in the expression. Each minicolumn of both algorithms participates in the expression of 273 inputs on average. With the increase of training rounds, SPL slowly decreases the standard deviation of the average participation degree due to the boosting rule and inhibition rule. This indicates that the utilization degree of the minicolumn becomes higher. For SPL_SN, the standard deviation of the average participation degree of the minicolumn is close to 0 in the first round of training, which means the proposed algorithm can efficiently utilize the minicolumns at the beginning.

#### 4.3.3. The New York Taxi Passenger Flow Dataset

The input space is constructed by the New York taxi passenger flow with 5000 data. In this dataset, the largest piece of data is 39375. Therefore, we set the code length to 40000 to express the input. This setting can meet the requirements of the input space encoding. The two algorithms train all data for 10 rounds. Then, according to the training results of each round, we evaluate the characteristics of the spatial pool.

**Figure 10:**
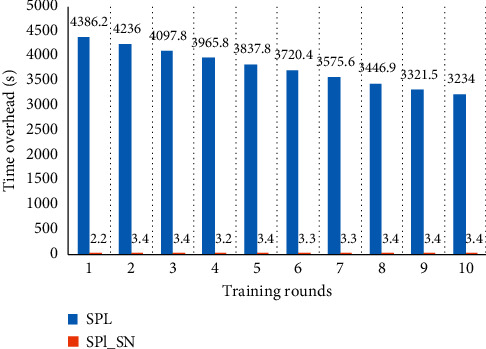
Comparison of training time overhead in the New York taxi passenger flow. Each column in the graph represents the training time overhead of the dataset after a round of training. The dataset was trained for 10 rounds using different algorithms.

Figure 10 shows the comparison of the training time overhead of each round. The time overhead of SPL is much larger than that of SPL_SN in each training round. It is because the maximum value of taxi passenger flow data is 39375, which requires a longer code to express the input. The longer code length increases the number of synapses of the minicolumn and the workload of calculating overlapping values. In SPL_SN, the input's compressed code is not affected by the code length, so the time overhead does not increase. These results are consistent with the prior experiment where the encoding has the same length.

**Figure 11:**
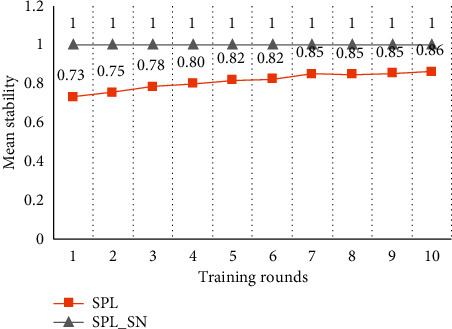
Comparison of stability in the New York taxi passenger flow. Each node in the graph represents the proportion of inputs whose SDRs have not changed in the whole dataset compared with the results of the previous round of training. The dataset was trained for 10 rounds using different algorithms.

Figure 11 shows the test results on the stability of the spatial pool. This result is the same as that of the artificial dataset with 5000 data. SPL_SN can keep the desired stability of the spatial pool. SPL increases the stability of the spatial pool with more training rounds, but ultimately it does not achieve the desired goal.

**Figure 12:**
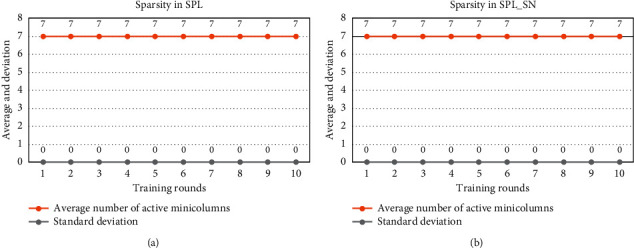
Comparison of sparsity in the New York taxi passenger flow. (a) After each round of TPL training, we calculate the mean and standard deviation of the number of activated minicolumns in each input to express the sparsity of the spatial pool. (b) After each round of TPL_SN training, we also count such indicators. The dataset was trained for 10 rounds using different algorithms.

Figure 12 shows the sparsity of the input representation. SPL_SN, like SPL, is able to express the input with a sparse minicolumn set according to the parameters.

**Figure 13:**
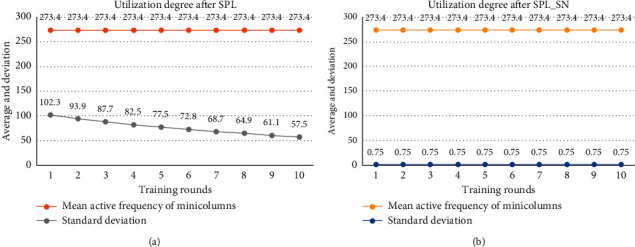
Comparison of the utilization degree of the minicolumn in the New York taxi passenger flow. (a) After each round of TPL training, we calculate the mean and standard deviation of the activation frequency of each minicolumn to express the utilization degree of the minicolumn. (b) After each round of TPL_SN training, we also count such indicators. The dataset was trained for 10 rounds using different algorithms.

Figure 13 shows the minicolumn utilization. This observation is the same as that of the artificial dataset with 5000 data. Both algorithms make efficient use of the minicolumns of the spatial pool, while SPL_SN achieves this faster.

## 5. Conclusions and Prospection

In this paper, we propose a fast spatial pool learning algorithm called SPL_SN. The spatial pool is reconstructed so that more information and states are provided into the minicolumn. We propose a minicolumn selection strategy based on the load-carrying degree and a synaptic adjustment algorithm based on compressed encoding. These improvements not only ensure the flexibility of the spatial pool but also greatly improve the training efficiency and stability. In the future research, we intend to construct a model based on HTM which can deal with the data with 3D (2D spatial+1D time) feature. Besides, we intend to implement a content-based fast retrieval system that can improve the retrieval speed of such applications.

## Figures and Tables

**Table 1:** The flow of SPL_SN.

(1) Encode the input (2) Calculate the overlapping value between the minicolumn and the input (3) Calculate the minicolumn's self-nomination status according to the overlapping value (4) Activate the minicolumns based on their self-nomination statuses and load-carrying capacity (5) Update the loading capacity and adjust synapses on activated minicolumns

**Table 2:** Parameter configuration for the spatial pool.

Common parameter	Value
COLUMN_DIMENSIONS	128
GLOBALINHIBITION	True
NUMACTIVECOLUMNSPERINHAREA	7.0
SYN_PERM_ACTIVE_INC	0.015
SYN_PERM_INACTIVE_DEC	0.0005
DUTY_CYCLE_PERIOD	1000
MAX_BOOST	10

## Data Availability

The artificial data used to support the findings of this study are included within the article. The time-period statistics of New York taxi passengers can be found at https://www1.nyc.gov/site/tlc/about/tlc-trip-record-data.page.
